# 4,4′-Dimethyl-2,2′-bipyridinium dichloride

**DOI:** 10.1107/S1600536808026615

**Published:** 2008-08-23

**Authors:** Urs David Eckensberger, Hans-Wolfram Lerner, Michael Bolte

**Affiliations:** a Institut für Anorganische Chemie, J. W. Goethe-Universität Frankfurt, Max-von-Laue-Strasse 7, 60438 Frankfurt/Main, Germany

## Abstract

In the title compound, C_12_H_14_N_2_^2+^·2Cl^−^, the 4,4′-dimethyl-2,2′-bipyridinium cation is essentially planar (r.m.s. deviation for all non-H atoms = 0.004 Å) and is located on a crystallographic inversion centre. The cations and chloride anions lie in planes parallel to (111) and are connected by N—H⋯Cl and C—H⋯Cl hydrogen bonds.

## Related literature

For related literature, see: Eckensberger (2006 ▶); Scheibitz *et al.* (2005 ▶). For structures containing the 4,4′-dimethyl-2,2′-bipyridinium cation, see: Linden *et al.* (1999 ▶); Willett *et al.* (2001 ▶).
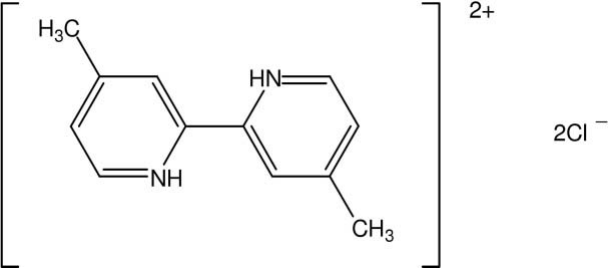


## Experimental

### 

#### Crystal data

C_12_H_14_N_2_^2+^·2Cl^−^
*M* *_r_* = 257.15
Triclinic, 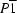

*a* = 5.1999 (10) Å
*b* = 7.2705 (13) Å
*c* = 8.4785 (15) Å
α = 93.877 (15)°
β = 102.349 (15)°
γ = 97.759 (15)°
*V* = 308.71 (10) Å^3^
*Z* = 1
Mo *K*α radiation
μ = 0.50 mm^−1^
*T* = 173 (2) K
0.21 × 0.21 × 0.14 mm

#### Data collection

Stoe IPDSII two-circle diffractometer
Absorption correction: multi-scan (*MULABS*; Spek, 2003 ▶; Blessing, 1995 ▶) *T*_min_ = 0.902, *T*_max_ = 0.933
3382 measured reflections
1147 independent reflections
926 reflections with *I* > 2σ(*I*)
*R*_int_ = 0.058

#### Refinement

*R*[*F* ^2^ > 2σ(*F* ^2^)] = 0.035
*wR*(*F* ^2^) = 0.079
*S* = 0.97
1147 reflections
78 parameters
H atoms treated by a mixture of independent and constrained refinement
Δρ_max_ = 0.23 e Å^−3^
Δρ_min_ = −0.23 e Å^−3^

### 

Data collection: *X-AREA* (Stoe & Cie, 2001 ▶); cell refinement: *X-AREA*; data reduction: *X-AREA*; program(s) used to solve structure: *SHELXS97* (Sheldrick, 2008 ▶); program(s) used to refine structure: *SHELXL97* (Sheldrick, 2008 ▶); molecular graphics: *XP* in *SHELXTL-Plus* (Sheldrick, 2008 ▶); software used to prepare material for publication: *SHELXL97*.

## Supplementary Material

Crystal structure: contains datablocks I, global. DOI: 10.1107/S1600536808026615/bi2297sup1.cif

Structure factors: contains datablocks I. DOI: 10.1107/S1600536808026615/bi2297Isup2.hkl

Additional supplementary materials:  crystallographic information; 3D view; checkCIF report

## Figures and Tables

**Table 1:** Hydrogen-bond geometry (Å, °)

*D*—H⋯*A*	*D*—H	H⋯*A*	*D*⋯*A*	*D*—H⋯*A*
N1—H1⋯Cl1	0.86 (3)	2.17 (3)	3.009 (2)	165 (3)
C2—H2⋯Cl1^i^	0.95	2.75	3.496 (2)	136
C5—H5⋯Cl1^ii^	0.95	2.62	3.554 (2)	169

Symmetry codes: (i) 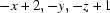; (ii) 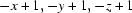.

**Fig. 1.**
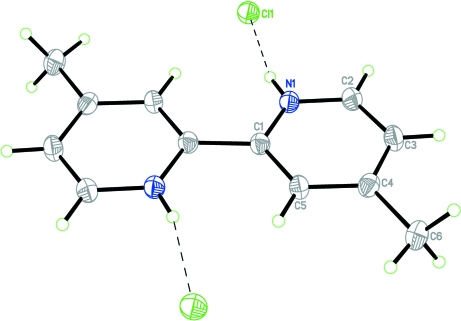
Perspective view of the title compound with the atom numbering scheme; displacement ellipsoids are at the 50% probability level; H atoms are drawn as small spheres of arbitrary radii. Hydrogen bonds are drawn as dashed lines. Symmetry operator for generating equivalent atoms: 1 - x, 1 - y, 1 - z.

**Fig. 2.**
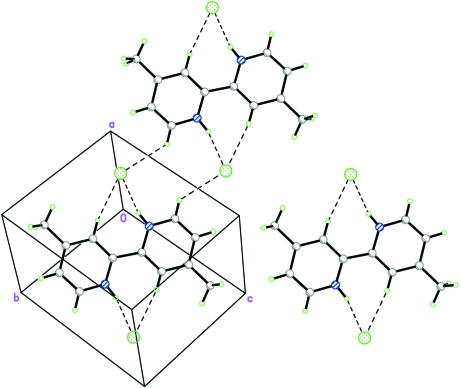
Packing diagram of the title compound viewed perpendicular to the (1 1 1) plane. Hydrogen bonds are indicated as dashed lines.

**Fig. 3.**
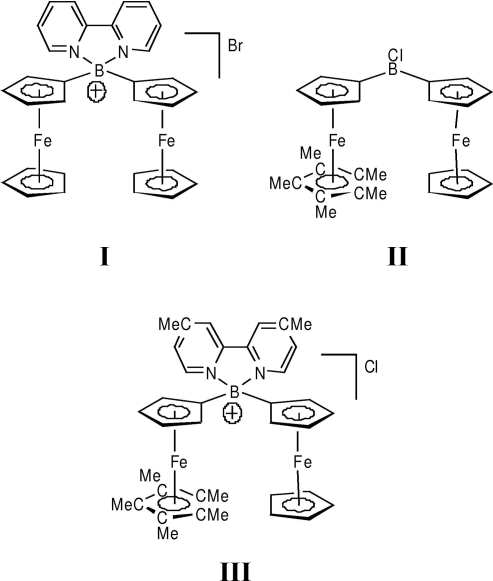
Reaction scheme.

## supplementary crystallographic information

### Comment

Recently, we have synthesized the dimeric diferrocenylboryl cation I (see Fig. 3) (Scheibitz *et al.*, 2005). Now we are interested to prepare the cationic dinuclear complex with a pentamethylcyclopentadienyl ring III. In an attempt to synthesize III from II (Eckensberger, 2006) and 4,4'-dimethyl-2,2'-bipyridine, we obtained the title compound as a by-product. X-ray quality crystals were grown from CD_3_CN in an NMR tube at ambient temperature.

The title compound crystallizes with one formula unit in the unit cell. The cation is located on a crystallographic inversion centre. It is essentially planar (r.m.s. deviation for all non-H atoms 0.004 Å). The chloride anions deviate by just 0.072 (3) Å from this plane. These planes are parallel to the (111) plane. In a plane, cations and anions are connected by N—H···Cl and C—H···Cl hydrogen bonds (Fig. 2).

### Experimental

In an attempt to synthesize complex III (Eckensberger, 2006) from II (0.156 g, 0.32 mmol) with 4,4'-dimethyl-2,2'-bipyridine (0.065 g, 0.35 mmol) in 5 ml acetonitrile, the title compound was obtained as a by-product. X-ray quality crystals were grown from CD_3_CN in an NMR tube at ambient temperature after several days.

### Refinement

H atoms were geometrically positioned with C_aromatic_—H = 0.95 Å and C_methyl_—H 0.98 Å, and refined using a riding model with *U*_iso_(H) = 1.2 *U*_eq_(C) or 1.5 *U*_eq_(C_methyl_)]. The methyl group was allowed to rotate about its local threefold axis. The H atom bonded to N was freely refined.

### Crystal data

**Table:**

C_12_H_14_N_2_^2+^·2(Cl^−^)	*Z* = 1
*M**_r_* = 257.15	*F*(000) = 134
Triclinic, *P*1	*D*_x_ = 1.383 Mg m^−^^3^
Hall symbol: -P 1	Mo *K*α radiation, λ = 0.71073 Å
*a* = 5.1999 (10) Å	Cell parameters from 3157 reflections
*b* = 7.2705 (13) Å	θ = 3.6–25.8°
*c* = 8.4785 (15) Å	µ = 0.50 mm^−^^1^
α = 93.877 (15)°	*T* = 173 K
β = 102.349 (15)°	Block, colourless
γ = 97.759 (15)°	0.21 × 0.21 × 0.14 mm
*V* = 308.71 (10) Å^3^	

### Data collection

**Table:**

Stoe IPDSII two-circle diffractometer	1147 independent reflections
Radiation source: fine-focus sealed tube	926 reflections with *I* > 2σ(*I*)
graphite	*R*_int_ = 0.058
ω scans	θ_max_ = 25.6°, θ_min_ = 3.6°
Absorption correction: multi-scan (*MULABS*; Spek, 2003; Blessing, 1995)	*h* = −6→6
*T*_min_ = 0.902, *T*_max_ = 0.933	*k* = −8→8
3382 measured reflections	*l* = −10→9

### Refinement

**Table:**

Refinement on *F*^2^	Primary atom site location: structure-invariant direct methods
Least-squares matrix: full	Secondary atom site location: difference Fourier map
*R*[*F*^2^ > 2σ(*F*^2^)] = 0.035	Hydrogen site location: inferred from neighbouring sites
*wR*(*F*^2^) = 0.079	H atoms treated by a mixture of independent and constrained refinement
*S* = 0.97	*w* = 1/[σ^2^(*F*_o_^2^) + (0.0407*P*)^2^] where *P* = (*F*_o_^2^ + 2*F*_c_^2^)/3
1147 reflections	(Δ/σ)_max_ < 0.001
78 parameters	Δρ_max_ = 0.23 e Å^−^^3^
0 restraints	Δρ_min_ = −0.23 e Å^−^^3^

### Special details

**Table:**

Geometry. All e.s.d.'s (except the e.s.d. in the dihedral angle between two l.s. planes) are estimated using the full covariance matrix. The cell e.s.d.'s are taken into account individually in the estimation of e.s.d.'s in distances, angles and torsion angles; correlations between e.s.d.'s in cell parameters are only used when they are defined by crystal symmetry. An approximate (isotropic) treatment of cell e.s.d.'s is used for estimating e.s.d.'s involving l.s. planes.
Refinement. Refinement of *F*^2^ against ALL reflections. The weighted *R*-factor *wR* and goodness of fit *S* are based on *F*^2^, conventional *R*-factors *R* are based on *F*, with *F* set to zero for negative *F*^2^. The threshold expression of *F*^2^ > σ(*F*^2^) is used only for calculating *R*-factors(gt) *etc*. and is not relevant to the choice of reflections for refinement. *R*-factors based on *F*^2^ are statistically about twice as large as those based on *F*, and *R*- factors based on ALL data will be even larger.

### Fractional atomic coordinates and isotropic or equivalent isotropic displacement parameters (Å^2^)

**Table:**

	*x*	*y*	*z*	*U*_iso_*/*U*_eq_	
Cl1	0.97517 (12)	0.22553 (7)	0.26756 (7)	0.02679 (18)	
N1	0.6763 (4)	0.3002 (2)	0.5264 (2)	0.0219 (4)	
H1	0.749 (6)	0.296 (4)	0.444 (4)	0.047 (8)*	
C1	0.5194 (4)	0.4274 (2)	0.5570 (2)	0.0195 (4)	
C2	0.7255 (5)	0.1636 (3)	0.6223 (3)	0.0254 (5)	
H2	0.8362	0.0774	0.5967	0.031*	
C3	0.6195 (5)	0.1455 (3)	0.7564 (3)	0.0273 (5)	
H3	0.6568	0.0483	0.8232	0.033*	
C4	0.4553 (4)	0.2725 (3)	0.7936 (3)	0.0223 (5)	
C5	0.4078 (4)	0.4121 (3)	0.6904 (2)	0.0210 (5)	
H5	0.2957	0.4988	0.7125	0.025*	
C6	0.3345 (5)	0.2555 (3)	0.9383 (3)	0.0287 (5)	
H6A	0.2337	0.3585	0.9488	0.043*	
H6B	0.4761	0.2605	1.0362	0.043*	
H6C	0.2147	0.1365	0.9244	0.043*	

### Atomic displacement parameters (Å^2^)

**Table:**

	*U*^11^	*U*^22^	*U*^33^	*U*^12^	*U*^13^	*U*^23^
Cl1	0.0285 (3)	0.0269 (3)	0.0288 (3)	0.00927 (19)	0.0113 (2)	0.00399 (18)
N1	0.0236 (10)	0.0228 (8)	0.0223 (10)	0.0070 (7)	0.0082 (8)	0.0064 (7)
C1	0.0204 (11)	0.0195 (9)	0.0181 (10)	0.0029 (8)	0.0031 (8)	0.0028 (8)
C2	0.0262 (12)	0.0238 (9)	0.0293 (12)	0.0086 (8)	0.0081 (10)	0.0084 (8)
C3	0.0284 (13)	0.0244 (10)	0.0295 (12)	0.0066 (9)	0.0032 (10)	0.0110 (9)
C4	0.0231 (11)	0.0217 (9)	0.0204 (10)	−0.0012 (8)	0.0035 (9)	0.0039 (8)
C5	0.0243 (12)	0.0196 (9)	0.0200 (10)	0.0061 (8)	0.0043 (9)	0.0049 (8)
C6	0.0350 (14)	0.0301 (11)	0.0218 (11)	0.0040 (10)	0.0076 (10)	0.0075 (9)

### Geometric parameters (Å, °)

**Table:**

N1—C2	1.342 (3)	C3—H3	0.950
N1—C1	1.360 (2)	C4—C5	1.397 (3)
N1—H1	0.86 (3)	C4—C6	1.498 (3)
C1—C5	1.382 (3)	C5—H5	0.950
C1—C1^i^	1.484 (4)	C6—H6A	0.980
C2—C3	1.372 (3)	C6—H6B	0.980
C2—H2	0.950	C6—H6C	0.980
C3—C4	1.404 (3)		
			
C2—N1—C1	121.9 (2)	C5—C4—C3	117.6 (2)
C2—N1—H1	113.5 (19)	C5—C4—C6	121.92 (17)
C1—N1—H1	124.6 (19)	C3—C4—C6	120.46 (19)
N1—C1—C5	118.08 (18)	C1—C5—C4	121.78 (17)
N1—C1—C1^i^	117.0 (2)	C1—C5—H5	119.1
C5—C1—C1^i^	124.9 (2)	C4—C5—H5	119.1
N1—C2—C3	121.46 (17)	C4—C6—H6A	109.5
N1—C2—H2	119.3	C4—C6—H6B	109.5
C3—C2—H2	119.3	H6A—C6—H6B	109.5
C2—C3—C4	119.17 (19)	C4—C6—H6C	109.5
C2—C3—H3	120.4	H6A—C6—H6C	109.5
C4—C3—H3	120.4	H6B—C6—H6C	109.5
			
C2—N1—C1—C5	−0.5 (3)	C2—C3—C4—C6	179.4 (2)
C2—N1—C1—C1^i^	179.7 (2)	N1—C1—C5—C4	0.9 (3)
C1—N1—C2—C3	0.0 (3)	C1^i^—C1—C5—C4	−179.3 (2)
N1—C2—C3—C4	0.2 (3)	C3—C4—C5—C1	−0.7 (3)
C2—C3—C4—C5	0.1 (3)	C6—C4—C5—C1	−180.0 (2)

Symmetry codes: (i) −*x*+1, −*y*+1, −*z*+1.

### Hydrogen-bond geometry (Å, °)

**Table:**

*D*—H···*A*	*D*—H	H···*A*	*D*···*A*	*D*—H···*A*
N1—H1···Cl1	0.86 (3)	2.17 (3)	3.009 (2)	165 (3)
C2—H2···Cl1^ii^	0.95	2.75	3.496 (2)	136
C5—H5···Cl1^i^	0.95	2.62	3.554 (2)	169

Symmetry codes: (ii) −*x*+2, −*y*, −*z*+1; (i) −*x*+1, −*y*+1, −*z*+1.
